# Supplementary material for: C-Reactive Protein/Albumin Ratio vs. Prognostic Nutritional Index as the Best Predictor of Early Mortality in Hospitalized Older Patients, Regardless of Admitting Diagnosis
Source: Nutrients. 2025 Sep 8;17(17):2907. doi: 10.3390/nu17172907 (PMC12430499; doi:10.3390/nu17172907)
Supplement: Supplementary file 1 [file nutrients-17-02907-s001.zip › nutrients-3788179-supplementary.pdf]

nutrients

MDPI

Supplementary Materials

TRIPOD Checklist: Prediction Model Development

Section/Topic	Item	Checklist Item	Paragraph
**Title and abstract**			
Title	1	Identify the study as developing and/or validating a multivariable prediction model, the target population, and the outcome to be predicted.	Title
Abstract	2	Provide a summary of objectives, study design, setting, participants, sample size, predictors, outcome, statistical analysis, results, and conclusions.	Abstract
**Introduction**			
Background and objectives	3a	Explain the medical context (including whether diagnostic or prognostic) and rationale for developing or validating the multivariable prediction model, including references to existing models.	Introduction, Background
	3b	Specify the objectives, including whether the study describes the development or validation of the model or both.	Introduction, last paragraph
**Methods**			
Source of data	4a	Describe the study design or source of data (e.g., randomized trial, cohort, or registry data), separately for the development and validation data sets, if applicable.	Methods, 2.1 Study Population
	4b	Specify the key study dates, including start of accrual; end of accrual; and, if applicable, end of follow-up.	Methods, 2.1 Study Population
Participants	5a	Specify key elements of the study setting (e.g., primary care, secondary care, general population) including number and location of centres.	Methods, 2.1 Study Population
	5b	Describe eligibility criteria for participants.	Methods, 2.1 Study Population
	5c	Give details of treatments received, if relevant.	Not relevant
Outcome	6a	Clearly define the outcome that is predicted by the prediction model, including how and when assessed.	Methods, 2.2 Data Collection
	6b	Report any actions to blind assessment of the outcome to be predicted.	Not applicable (retrospective study)
Predictors	7a	Clearly define all predictors used in developing or validating the multivariable prediction model, including how and when they were measured.	Methods, 2.2 Data Collection
	7b	Report any actions to blind assessment of predictors for the outcome and other predictors.	Not applicable (retrospective study)

Sample size	8	Explain how the study size was arrived at.	Methods, 2.1 Study Population
Missing data	9	Describe how missing data were handled (e.g., complete-case analysis, single imputation, multiple imputation) with details of any imputation method.	Methods (exclusion criteria)
Statistical analysis methods	10a	Describe how predictors were handled in the analyses.	Methods, 2.3 Statistical Analysis
	10b	Specify type of model, all model-building procedures (including any predictor selection), and method for internal validation.	Methods, 2.3 Statistical Analysis
	10c	Specify all measures used to assess model performance and, if relevant, to compare multiple models.	Methods, 2.3 Statistical Analysis
Risk groups	11	Provide details on how risk groups were created, if done.	Not done
**Results**			
Participants	13a	Describe the flow of participants through the study, including the number of participants with and without the outcome and, if applicable, a summary of the follow-up time. A diagram may be helpful.	Results, 3.1 + Flow Chart
	13b	Describe the characteristics of the participants (basic demographics, clinical features, available predictors), including the number of participants with missing data for predictors and outcome.	Results, 3.1 General Characteristics
Model development	14a	Specify the number of participants and outcome events in each analysis.	Results, 3.4 Mortality
	14b	If done, report the unadjusted association between each candidate predictor and outcome.	Results, 3.4 Mortality
Model specification	15a	Present the full prediction model to allow predictions for individuals (i.e., all regression coefficients, and model intercept or baseline survival at a given time point).	Results, 3.4 Predictive performance
	15b	Explain how to the use the prediction model.	Results, 3.4 Predictive performance
Model performance	16	Report performance measures (with CIs) for the prediction model.	Results, 3.4 Predictive performance
**Discussion**			
Limitations	18	Discuss any limitations of the study (such as nonrepresentative sample, few events per predictor, missing data).	Discussion, last paragraphs
Interpretation	19b	Give an overall interpretation of the results, considering objectives, limitations, and results from similar studies, and other relevant evidence.	Discussion, entire section
Implications	20	Discuss the potential clinical use of the model and implications for future research.	Discussion, last paragraphs
**Other information**			

Supplementary information	21	Provide information about the availability of supplementary resources, such as study protocol, Web calculator, and data sets.	Institutional Review Board Statement section
Funding	22	Give the source of funding and the role of the funders for the present study.	Funding section

Figure S1. Calibration plot concerning the 7-day predictive power of death of the CRP/Alb ratio (performed with STATA).

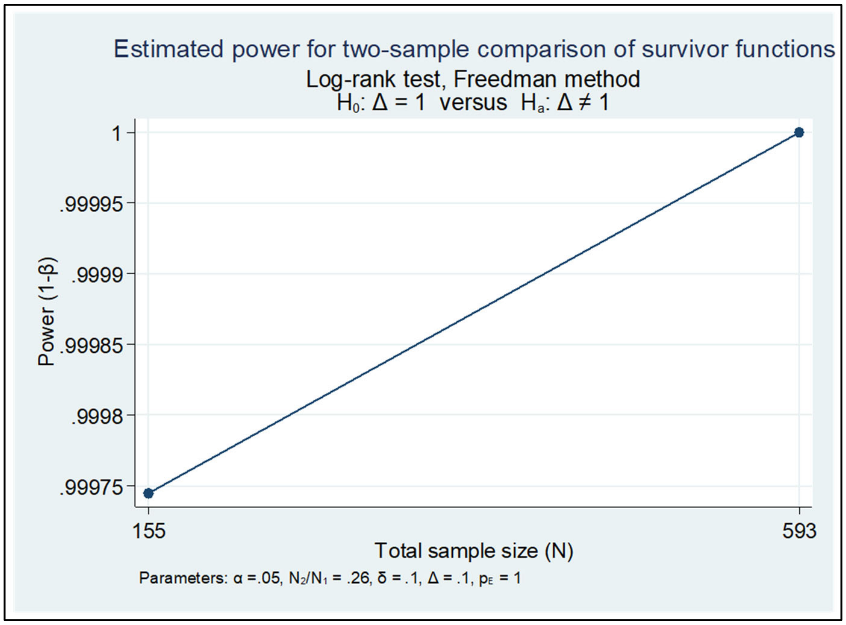

Figure S2. Calibration plot concerning the 7-day predictive power of death of the PNI (performed with STATA).

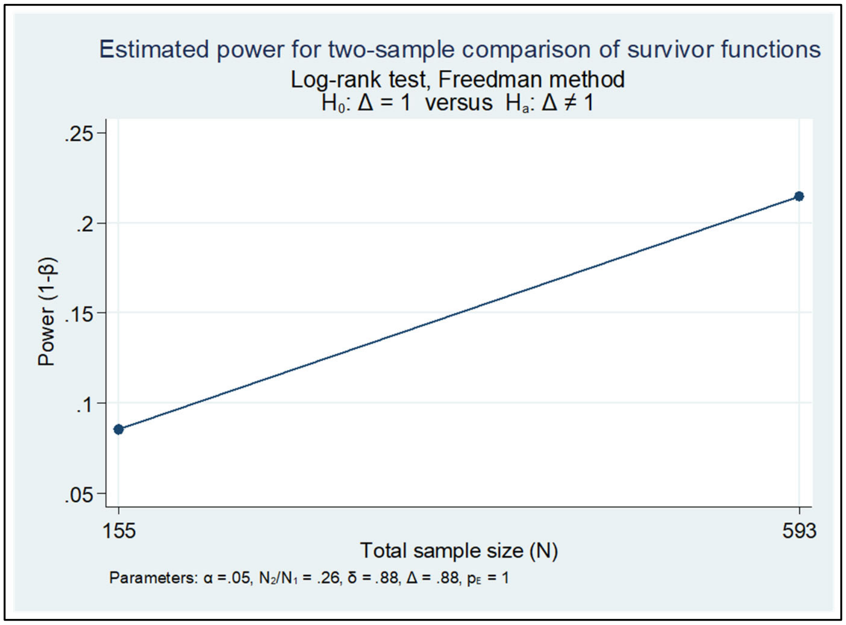

Figure S3. Calibration plot concerning the 30-day predictive power of death of the CRP/Alb ratio (performed with STATA).

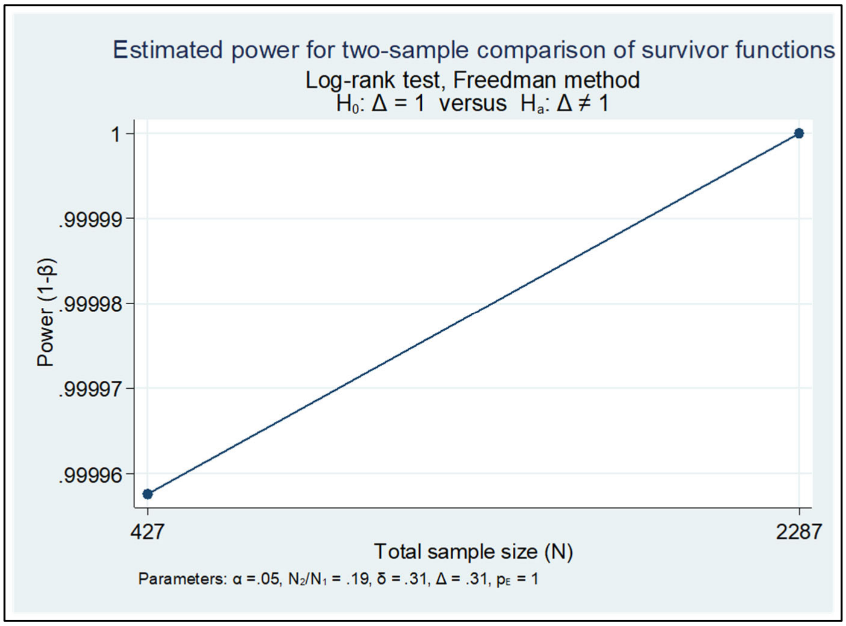

Figure S4. Calibration plot concerning the 30-day predictive power of death of the PNI (performed with STATA).

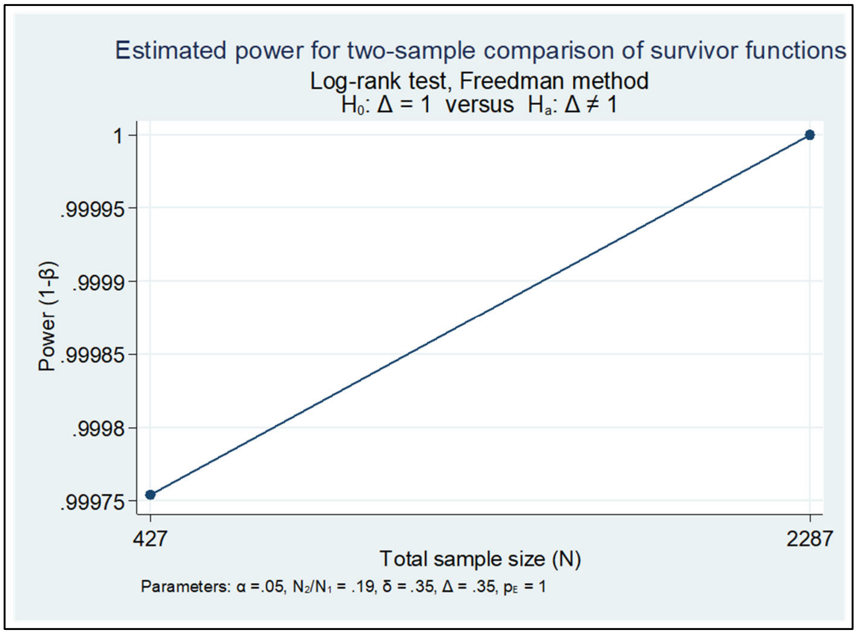

Table S1. Hosmer–Lemeshow goodness-of-fit test both PNI and CRP/Alb ratio, 7 days vs 30 days

	7 Days	30 Days
**Number of observations**	748	2714
**Number of covariate patterns**	4	4
**Pearson chi2(1)**	0,98	2,74
**P value**	0,32	0,09
